# Division of overall duration of stay into operative stay and postoperative stay improves the overall estimate as a measure of quality of outcome in burn care

**DOI:** 10.1371/journal.pone.0174579

**Published:** 2017-03-31

**Authors:** Islam Abdelrahman, Moustafa Elmasry, Pia Olofsson, Ingrid Steinvall, Mats Fredrikson, Folke Sjoberg

**Affiliations:** 1 Plastic Surgery Unit, Surgery Department, Suez Canal University, Ismailia, Egypt; 2 Department of Plastic Surgery, Hand Surgery, and Burns, Linköping University, Linköping, Sweden; 3 Department of Clinical and Experimental Medicine, Linköping University, Linköping, Sweden; 4 Department of Anaesthesiology and Intensive Care, Linköping University, Linköping, Sweden; Association of Dutch Burn Centres, NETHERLANDS

## Abstract

Total duration of stay adjusted for percentage of the total body surface area burned (TBSA%) is a commonly used outcome measure in burn care. However, it has been criticised as it is affected by many factors, some of which are not strictly part of burn care. A division into operative stay and postoperative stay may improve this measure. The aim was to evaluate if operative stay can serve as a more standardised measure by: comparing the variation in operative stay/TBSA% with the variation in total stay/TBSA%, and to study different factors associated with operative stay and postoperative stay.

**Patients and methods:**

Surgically managed burn patients admitted between 2010–14 were included. Operative stay was defined as the time from admission until the last operation, postoperative stay as the time from the last operation until discharge. The difference in variation was analysed with F-test. A retrospective review of medical records was done to explore reasons for extended postoperative stay. Multivariable regression was used to assess factors associated with operative stay and postoperative stay.

**Results:**

Operative stay/TBSA% showed less variation than total duration/TBSA% (F test = 2.38, p<0.01). The size of the burn, and the number of operations, were the independent factors that influenced operative stay (R^2^ 0.65). Except for the size of the burn other factors were associated with duration of postoperative stay: wound related, psychological and other medical causes, advanced medical support, and accommodation arrangements before discharge, of which the two last were the most important with an increase of (mean) 12 and 17 days (p<0.001, R^2^ 0.51).

**Conclusion:**

Adjusted operative stay showed less variation than total hospital stay and thus can be considered a more accurate outcome measure for surgically managed burns. The size of burn and number of operations are the factors affecting this outcome measure.

## Introduction

Measures for the evaluation of the outcome of care of burns have evolved over time starting with mortality [1], followed by duration of hospital stay [2], and ending up with quality of life measures and assessments of scars [3]. In our Burn Centre, total duration of stay was used for a long time as an important measure of outcome [4]. However, there were several drawbacks, mainly concerning differences between those patients managed surgically and those managed conservatively [5]. Adjustment of total duration of stay to percentage of total body surface area burned (TBSA%) is a more promising way to evaluate outcome [5–7], but the figures vary among centres and can also vary with different age groups and TBSA% [5, 8].

The absence of standard discharge criteria after inpatient treatment of a burn is also important, as it can be influenced by administrative policies and other logistic issues [2, 3, 5], and can result in longer stays. Many reasons for extended stay can be considered [2, 9–12]: first wound related, secondly need for advanced medical support and additional treatment for psychological or other medical causes, or accommodation preparation before discharge. The need for an outcome measure that is more reproducible than duration of total stay is evident.

**Fig 1:**
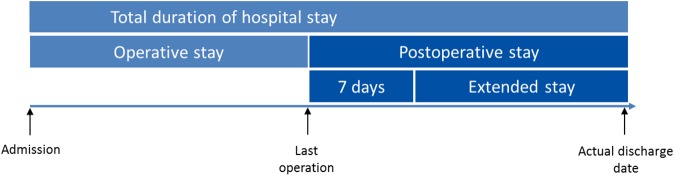
Outline of the new subdivisions. The operative duration of stay and the postoperative stay in burned patients treated operatively.

We are suggesting an improvement in the measurement of hospital stay for the care of burned patients who require operation, focusing on the surgical part of the period of care. In patients with deep burns that need excision and grafting the period between admission to the Burn Centre until all skin grafts are complete and the treatment is at an end is the core period of treatment. This can be followed by a further interval that we call the postoperative time. We assume that the time that a patient spends in hospital after the operation has been finished could indicate reasons that prolong the total time spent in hospital. We hypothesize that the division of total duration of stay into operative stay and postoperative stay may result in a more precise way of comparing outcome after burn care as the operative stay could be defined solely by surgical factors, and the postoperative stay could be affected by administrative and other factors. We therefore suggest two subdivisions of duration of stay: “Operative stay” which lasts from the day of admission until the last operation, and the “postoperative stay” which is the time from the last operation until discharge from the Burn Centre (Fig 1).

We are suggesting an improvement in the measurement of total duration of stay for the care of burned patients who required operation, focusing on the surgical part of the period of care.The aim of the present study was to evaluate if operative stay can serve as a more standardised measure by: comparing the variation in operative stay/TBSA% with the variation in total stay/TBSA%, and to study different factors associated with operative stay and postoperative stay.

## Patients and methods

In this retrospective clinical cohort study, all patients admitted to Linköping University Hospital Burn Centre between 2010 and 2014 were screened for eligibility. Inclusion criteria were: patients with burn injury that required surgical intervention, and who were alive at discharge. Exclusion criterion was: patients with minor burns who were admitted repeatedly for one day at a time (scattered stays).

All patients (adults, parents and guardians for minors) were informed verbally of the collection of data and had the opportunity to not contribute with data to the registry. The data was extracted from the burn centre database and the electronic medical journal system retrospectively under the protection of the county council in Linköping data network security system, all patients social security codes were coded to be unidentifiable. Analysis and registering of data afterwards was done unidentifiable. The study was approved by the Regional Ethics Review Board in Linköping (2013/341-31).

### Treatment

Patients were treated according to a fixed protocol, including early excision and grafting [13], revision of the wound every second day, standard ventilation when needed [14, 15], together with parenteral fluids [16] and early enteral nutrition. Those with minor burns had early tangential excision during the first 48 hours whereas most of the surface area was excised during the first operation for deep burns, which were covered by meshed split thickness skin grafts. Major burns were treated by staged excisions and covered with xenografts to allow clear demarcation of the wound’s bed. This was followed by the autograft with a meshed split thickness skin graft when the bed was ready [12, 17, 18].

### Data analysis

Variables studied were:

Demographic data: Age and sex

Those related to the burn: TBSA%, superficial dermal (%), deep dermal (%), full thickness burn (%), total duration of stay, postoperative stay, operative stay, operative stay/TBSA%, total duration of stay/TBSA%, and postoperative stay/total stay presented as percentage, cause of burn, reasons for extended postoperative stay, number of operations, duration of operation (minutes).

Administrative: Region of residence was divided into our region (the referral region for the Department of Plastic Surgery, Hand Surgery, and Burns) and those who were referred from outside that region (satellite patients).

### Description of the process of content qualitative analysis

As there are no previous studies describing reasons for extended postoperative stay a content qualitative analysis [19] was applied on all patients who stayed more than 7 days after the last operation. First the patients’ medical records were reviewed and possible reasons for extended stay were identified as content units, the aim was to answer the question “why are burned patients who are treated surgically kept in hospital for longer than 7 days after the last skin graft?” The next step was categorizing the content units into groups as follows: Wound related, Advanced medical support required, Accommodation arrangements, Psychological or other medical causes, No obvious cause (no reason given for the delay in discharge). The reasons were gathered and categorised independently by three of the authors and the final decision was taken by consensus.

We hypothesized that patients who made an uneventful recovery would stay in the unit for a maximum of seven days after the last operation. The calculation of seven days is based on the assumption that a skin graft is usually the last operation done, and the dressing is changed five days later, followed by one further dressing to exclude infections and failure of the graft [9, 20]. We then considered that patients who stayed for more than seven days had by definition extended their postoperative stay, which was the total of days beyond seven days after the last intervention (Fig 1).

### Statistical analysis

Data were analysed with the help of STATA (STATA v12.0, Stata Corp. LP College Station, TX, USA), and presented as median (10–90 centiles) unless otherwise stated. The significance of differences in characteristics were assessed with Mann Whitney *U* test and the chi square test, and one sample test of proportions based on the binomial distribution was used to test if the proportion of subgroups differed from 50%. The F test was used to assess the significance of the difference in variance between two variables (operative stay/TBSA% and total stay/TBSA%). The Kruskal-Wallis ANOVA was used for the analysis of multiple groups (TBSA% groups), and the Mann Whitney *U* test post hoc. Multivariable regression was used to analyse factors for operative stay and postoperative stay. The model was designed to cover: demographic aspects (age and sex); aspects of the burn injury (superficial dermal %, deep dermal %, full thickness burn %, cause of burn); burn treatment (number of operations, duration of operation); and the main causes for extended postoperative stay. All variables were included in the initial model. Linear correlation was used for multicollinearity test within the different aspects of the model, and in case of r values close to 1 the variable with less impact on model R^2^ was removed. We have presented all variables of the final model in the tables. Probabilities of less than 0.05 were accepted as significant.

## Results

### General description

**Fig 2:**
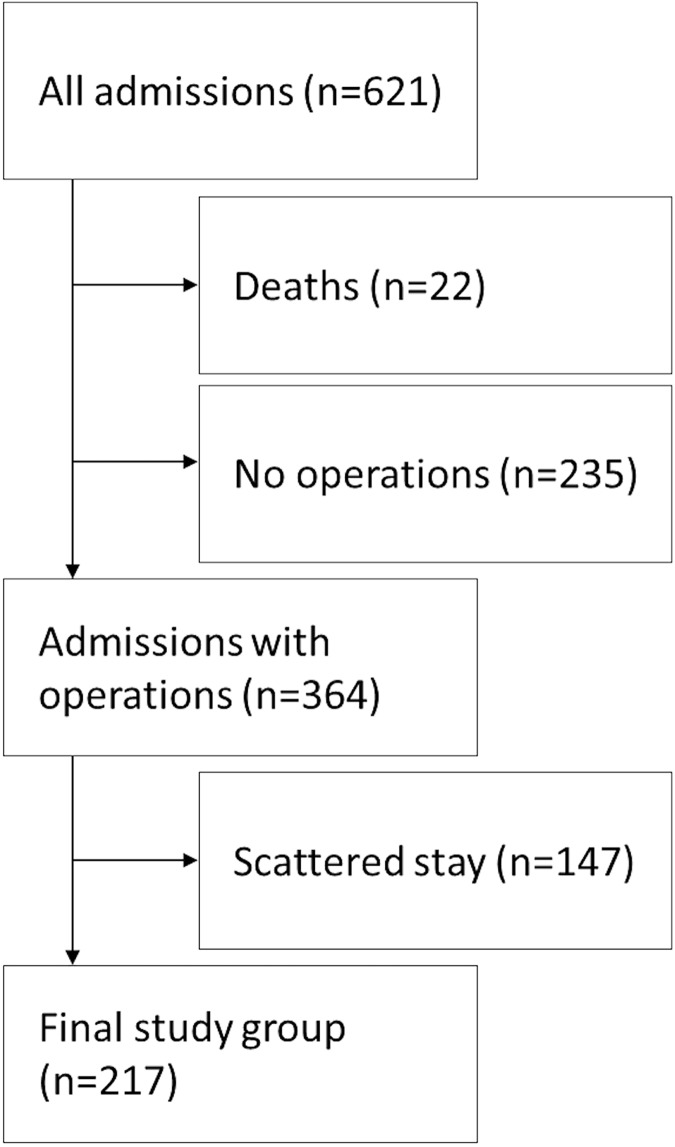
Selection of patients included.

A total of 217 patients were included (Fig 2). The study group was divided into those whose postoperative stay was extended and those for whom it was not, and the groups differed in baseline characteristics (Table 1) and treatment characteristics (Table 2). The patients with extended postoperative stay were older, had larger TBSA%, a higher proportion of flame burns, and a higher proportion of satellite patients (Table 1). Median operative stay/TBSA% for all patients was 0.9 days (Table 2).

**Table 1:** Baseline characteristics of the patients, grouped by extended postoperative stay.

	All	Not extended	Extended	P value
Patients, n	217	110 (51)	107 (49)	
Satellite patients, n (%)	143 (66)	60 (55)	83 (78)	<0.001
TBSA%	9.0 (1.5–36.0)	5.0 (1.1–20.5)	15.0 (3.5–49.5)	<0.001
Deep dermal and full thickness burn%	3.6 (0.0–28.5)	1.9 (0.0–14.3)	8.5 (1.0–41.5)	<0.001
Sex, men (%)	150 (69)	80 (73)	70 (65)	0.24
Age, years	44.0 (2.0–75.0)	37.5 (1.0–73.5)	47.0 (10.0–80.0)	0.02
Cause of injury				
Scalds	46	30 (65)	16 (35)	0.05
Chemical	9	8 (89)	1 (11)	0.04
Hot object	20	14 (70)	6 (30)	0.12
Electricity	13	7 (54)	6 (46)	1.00
Flame burns	129	51 (40)	78 (60)	0.03

Data are presented as median (10–90 centiles) or n (%). Mann Whitney U test and Chi square test as appropriate, and one sample test of proportions based on the binomial distribution for subgroup analyse (cause of injury).

**Table 2:** Treatment characteristics, grouped by extended postoperative stay.

	All	Not extended	Extended	P value
Patients, n	217	110 (51)	107 (49)	
Operative stay, days	10 (0–42)	3.5 (0–22.5)	16 (2–51)	<0.001
Postoperative stay, days	7.0 (1.0–15.0)	5.5 (1.0–7.0)	12.0 (8.0–22.0)	<0.001
Total stay, days	19.0 (3.0–58.0)	8.0 (1.0–29.0)	28.0 (14.0–76.0)	<0.001
Operative stay, %	55 (0–83)	50 (0–88)	57 (13–79)	0.06
Postoperative stay, %	44 (15–100)	44 (9–100)	43 (21–88)	0.24
Operative stay/TBSA%	0.9 (0–2.9)	0.7 (0–2.9)	1.1 (0.3–2.8)	<0.001
Total stay/TBSA%	2.0 (0.7–5.6)	1.7 (0.6–5.6)	2.1 (1.0–5.4)	0.03
Operations, n	2.0 (1.0–8.0)	1.0 (1.0–4.0)	3.0 (1.0–9.0)	<0.001
Total operation time, minutes	120.0 (60.0–1320.0)	120.0 (60.0–480.0)	300.0 (60.0–1620.0)	<0.001
Operation minutes/TBSA%	26.7 (6.7–80.0)	26.7 (7.4–96.2)	28.8 (5.5–64.3)	0.68

Data are presented as median (10–90 centiles) or n (%). Mann Whitney U test. Operative stay % is the percentage of operative stay out of the total duration of hospital stay. Postoperative stay % is the percentage of postoperative days out of the total duration of hospital stay.

### Operative stay/TBSA% compared with total stay /TBSA%

**Fig 3:**
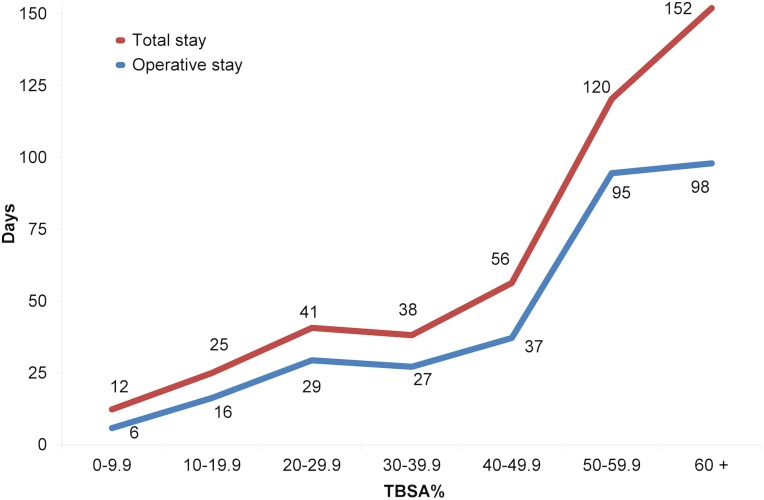
Mean total stay and operative stay by TBSA% groups.

**Fig 4:**
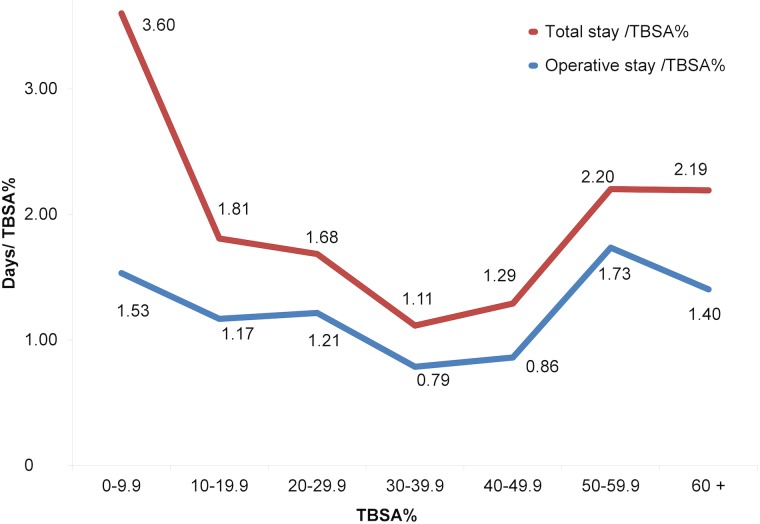
Mean total stay/TBSA% and operative stay/TBSA% by TBSA% groups.

Operative stay/TBSA% (mean 1.4 days/TBSA%, 95% CI 1.1 to 1.6, variance 2.9) showed a smaller variation among the studied population when comparing with total stay/TBSA% (mean 2.7 days/TBSA%, 95% CI 2.4 to 3.1, variance 6.9) (F test = 2.38, p<0.01). The difference between mean total stay and operative stay was remarkable (54 days, 95% CI -46 to 154 days) among the group with TBSA% of 60% or more (Fig 3). The total stay/TBSA% was longest in both the group with the smallest and the largest TBSA%, while the operative stay/TBSA% was almost constant (Fig 4). There was no difference in operative stay/TBSA% between the TBSA% groups (p = 0.64), but there was a highly significant difference in total stay/TBSA% between the TBSA% groups (p<0.001). Post hoc analysis showed that this difference mainly resulted from the higher ratio in the group with the smallest TBSA% (p<0.01 in the groups with TBSA% <50%).

### Description of the patients grouped by reasons for extended postoperative stay

**Fig 5:**
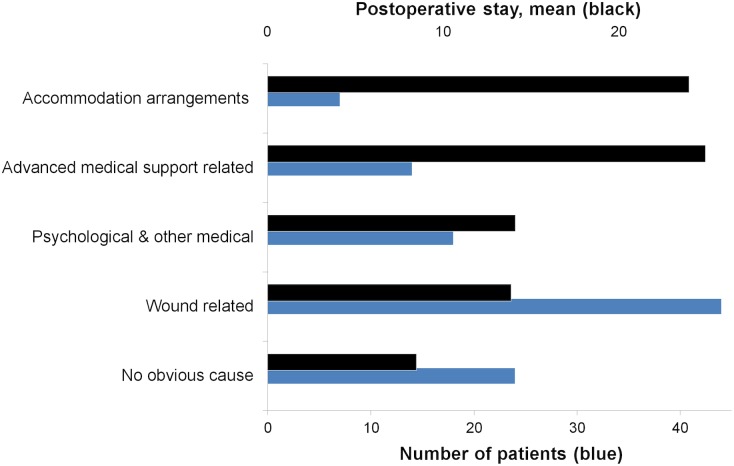
Mean postoperative stays (days) for each cause group.

**Fig 6:**
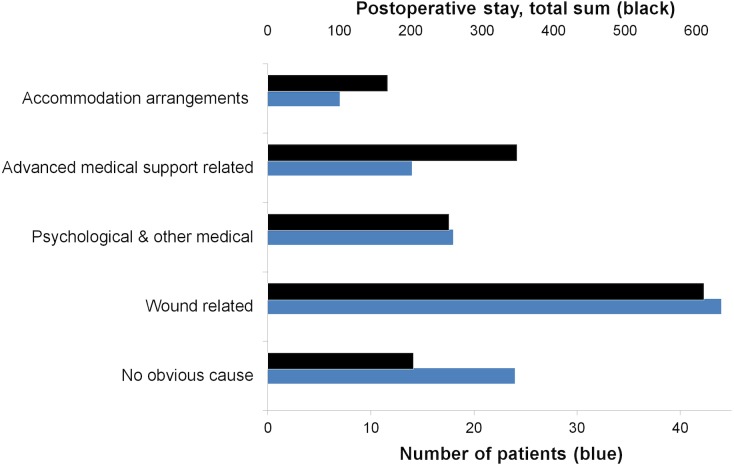
Total sum of postoperative stays (days) for each cause group.

Among the 107 patients whose postoperative stay was extended, wound-related problems constituted 41% (44/107) with a median postoperative stay of 12 days. The longest postoperative stays were among the groups; Advanced medical support, and Accommodation arrangements. Although the number of patients with no obvious reason was high (22/107), the median postoperative stay in this group was the shortest (Table 3). Mean postoperative stays and number of patients are shown in Figs 5 and 6.

**Table 3:** Details of the patients by extended postoperative stay cause group.

	Patients	Satellite patients	Age, years	Sex, men	TBSA%	Postoperative stay	Total stay	Total stay /TBSA%	Operations
Wound related	44 (41)	35 (80)	42 (14–71)	28 (64)	17.5 (4.8–55)	12 (9–21)	26.5 (14–63)	1.7 (0.9–4.0)	2 (1–8)
Psychological & other medical	18 (17)	12 (67)	40.5 (2–66)	11 (61)	16.5 (2–53)	12 (9–20)	26.5 (13–76)	2.0 (1.0–8.2)	3 (1–8)
Advanced medical support related	14 (13)	9 (64)	57.5 (0.5–87)	9 (64)	26.3 (4–62)	14.5 (12–70)	50.5 (23–226)	3.0 (1.3–6.1)	7.5 (1–16)
Accommodation arrangements	7 (7)	7 (100)	51 (2–94)	4 (57)	11 (2.5–45.3)	14 (8–81)	27 (14–95)	2.3 (1.8–5.6)	2 (1–9)
No obvious cause	24 (22)	20 (83)	52.5 (13–72)	18 (75)	9.3 (3–29.3)	8 (8–10)	26.5 (15–47)	2.2 (1.2–5.2)	3 (1–5)
Total	107	83 (78)	47 (10–80)	70 (65)	15 (3.5–49.5)	12 (8–22)	28 (14–76)	2.1 (1–5.4)	3 (1–9)

Data are presented as median (10–90 centiles) or n (%).

### Factors that affected operative stay

Number of operations and the extent of the burn were independent factors for duration of operative stay. Neither age, sex, nor cause of injury contributed significantly to the model. The variable “duration of operation” was removed from the model as it was intercorrelated with the number of operations (r = 0.97). Operative stay was not confounded by the main reasons for extended postoperative stay except for Advanced medical support required, although we did not study enough patients to achieve significance (Table 4). S1 Table shows that operative duration of stay can be predicted by the extent and depth of the burn injury with a model R^2^ of 0.56.

**Table 4:** Multivariable regression for operative stay.

	Coefficient	p value	95% CI
Superficial dermal %	0.40	0.04	0.03 to 0.77
Deep dermal %	0.08	0.59	-0.21 to 0.36
Full thickness burn %	1.06	<0.001	0.67 to 1.45
Operations, number	4.35	<0.001	2.95 to 5.75
Age (years)	-0.06	0.28	-0.16 to 0.05
Sex (male)	-1.80	0.49	-6.90 to 3.30
Cause of burn			
Scalds			
Chemical	3.73	0.56	-8.89 to 16.35
Hot object	2.12	0.65	-7.09 to 11.33
Electricity	-0.74	0.89	-11.77 to 10.28
Flame	3.18	0.36	-3.60 to 9.95
Main causes for extended postoperative stay*			
No obvious cause	3.61	0.36	-4.11 to 11.32
Wound related	-3.83	0.26	-10.44 to 2.79
Psychological & other medical	-3.70	0.41	-12.47 to 5.06
Advanced medical support related	10.59	0.05	-0.20 to 21.38
Accommodation arrangements	-7.57	0.26	-20.82 to 5.69
Constant	-1.18	0.74	-8.18 to 5.82

Model adjusted R^2^ 0.65 p<0.001, n = 217.

*Reference group was the 110 patients who not had extended postoperative stay.

### Factors that affected postoperative stay

Longer postoperative stay was associated with the extent of the burn and all of the main causes for extended postoperative stay except for the group with “no obvious cause”. The groups with the longest postoperative stays were the ones with issues related to accommodation arrangements (mean 16.9 days longer) and advanced medical support (mean 11.8 days longer). (Table 5).

**Table 5:** Multivariable regression for postoperative stay.

	Coefficient	p value	95% CI
Superficial dermal %	0.05	0.54	-0.11 to 0.21
Deep dermal %	0.16	0.01	0.04 to 0.28
Full thickness burn %	0.29	0.001	0.12 to 0.46
Operations, number	0.57	0.07	-0.04 to 1.18
Age (years)	0.00	0.97	-0.05 to 0.04
Sex (male)	-0.16	0.88	-2.38 to 2.05
Cause of burn			
Scalds			
Chemical	0.19	0.95	-5.28 to 5.66
Hot object	0.82	0.69	-3.17 to 4.81
Electricity	-2.49	0.31	-7.27 to 2.29
Flame	0.01	1.00	-2.93 to 2.95
Main causes for extended postoperative stay*			
No obvious cause	1.94	0.25	-1.40 to 5.29
Wound related	5.54	<0.001	2.67 to 8.40
Psychological & other medical	6.57	0.001	2.77 to 10.37
Advanced medical support related	11.80	<0.001	7.12 to 16.48
Accommodation arrangements	16.86	<0.001	11.12 to 22.61
Constant	2.44	0.11	-0.59 to 5.48

Model adjusted R^2^ 0.51 p<0.001, n = 217.

*Reference group was the 110 patients who not had extended postoperative stay.

The main causes for extended postoperative stay accounted for 13% of the model strength (removing this categorised variable showed a decrease of adjusted R^2^ from 0.51 to 0.38), while the size of the burn (percentage superficial dermal, deep dermal, and full thickness burn) accounted for 3% of the total model (a decreased of adjusted R^2^ from 0.51 to 0.48).

## Discussion

This is to our knowledge the first study to focus on duration of hospital stay (in patients whose burns were treated surgically) during the core period of surgical treatment. We subdivided total duration of stay into two well-defined entities (operative stay, and postoperative stay), the cutoff point being the last autograft day. The use of this day as cutoff seems arbitrary but regardless the size of the burn or the different surgical plans applied there is still always a last operation applying autografts, which generates a substantial degree of standardization. Variations in this period of the care should reflect the efficiency of the surgical plans in different burn centres more closely than the duration of total hospital stay does, which makes operative stay an interesting measure for benchmarking purposes.

We found that operative stay was a more consistent measure than total stay. The proposed new divisions of time periods can facilitate investigation of the underlying factors that affect both periods, and make it easier to study the reasons for long stays in hospital.

### Duration of operative stay/TBSA% compared with total stay/TBSA%

We found that operative stay/TBSA% was a reliable tool for judging the surgical care of burns with few confounders, even though it does not reflect the actual healing time. In the current study, almost half the patients had extended postoperative stays [3]. Healing time as an outcome measure could be an alternative to help to evaluate the surgical care of burns [10, 21, 22], but the difficulties of definition and estimation of the accurate healing time limits its use [2].

Total duration of stay/TBSA% is often criticised when it is used in patients with smaller TBSA% burns, as it leads to disproportionally high values compared with groups with larger TBSA% [5, 7]. This is in contrast to the results in the present study in which we use duration of operative stay/TBSA%, as it gave more consistent and comparable values.

### Reasons for extended postoperative stay

Issues related to accommodation and advanced medical support proved to be the strongest independent predictors for longer postoperative stays. Advanced medical support was the issue in only a few patients with extended postoperative stays (13%), but its impact in extending the duration of postoperative stay was large, which is in line with a previous study in which admission to the ICU was shown to be a strong predictor of longer overall duration of stay [2]. There were wound-related issues in 41% of the patients with extended postoperative stays, and their median was 12 days. This outlines the validity of the concept of operative stay/ postoperative stay as it could detect longer periods of care that can be reduced by transferring the patient back to his home hospital, or supplying the care in outpatients if the patient’s general condition permits [9].

### Factors that affected operative stay

As anticipated, extension and depth of the burn were strongly associated with the duration of operative stay. This strengthens the value of it as a predictor of surgical care and the high R^2^ value (0.56) in the corresponding model (S1 Table) supports the validity of the proposed concept of operative stay, and is in line with other studies that have analysed predictors for duration of stay [2]. Female sex was not an independent factor for longer operative stay, which is in line with some studies [11, 23, 24] but not others [2, 25–28].

Age was not a predictor for longer operative stays, which was not in line with most of the reported results of regression models for total duration of stay [23–27]. We think that the improved management of elderly patients during the last decade [29] can explain that age no longer is an independent factor for duration of stay.

### Factors that affected postoperative stay

Longer postoperative stay was associated with the extent of the burn and all except one of the main causes for extended postoperative stay. These results indicate that it can be interesting to investigate each period (operative stay and postoperative stay) separately in the future. The group with extended postoperative stays had more severe burns and subsequently a higher risk for organ dysfunction and other complications, and a higher need for advanced medical support. We did not include any organ function score in the current study, as it is somewhat outside the scope of the aim, but it would be interesting to compare the length of postoperative care between burn centres (adjusted for TBSA%) and comparing the development of organ dysfunctions and other complications among the patients who required prolonged stay for medical reasons.

### Limitations

One limitation is the variation among different burn centres in their approach to excision of burns, with different timings and different intervals, which could hinder the generalisation of the results. The use of the last operation as a cut-off point can be questioned for the same reason. However, the centres that achieve the shortest operative stay, regardless of timing, surgical management, or alternative wound management, would be able to show the direct benefit of their management with the use of the outcome measure “operative stay”.

Another limitation was that it was retrospective when looking for the reasons for extended postoperative stays, and it was difficult in some cases to retrieve a supposed cause of delayed discharge. Nevertheless all groups except one were represented in the regression model showing different impacts which can reflect the real situation for this care period. In the most difficult cases we preferred to add them to the “no obvious cause” group. However, this group had the shortest postoperative stays of all groups.

The exclusion of 147 patients because of the short stay policy may also be a limitation. However, this group of patients needed procedures that required only one day in hospital, so had no extended postoperative stay. Excluding or selecting short stay patients has previously been used by several authors [5].

## Conclusion

Adjusted operative stay showed less variation than total hospital stay and thus can be considered a more accurate outcome measure for surgically managed burns. The size of burn and number of operations are the factors affecting this outcome measure. The concept of postoperative care period can be used as an instrument to monitor the total duration of a patient’s hospital stay.

## Supporting information

S1 Table The predictive model for operative stay. A multivariable regression analysis showed that duration of operative stay can be predicted by the extent and depth of the burn injury. The three variables: superficial second degree %, deep second degree %, and full thickness burn %, gave a model adjusted R^2^ of 0.56.
(PDF)

S1 File Data spreadsheet. (XLSX)

## References

[1] Bull JP, Squire JR. A Study of Mortality in a Burns Unit: Standards for the Evaluation of Alternative Methods of Treatment. Ann Surg. 1949;130(2):160–73. Epub 1949/08/01. PubMed Central PMCID: PMC1616308. 17859418 10.1097/00000658-194908000-00002 PMC1616308

[2] Hussain A, Dunn KW. Predicting length of stay in thermal burns: A systematic review of prognostic factors. Burns. 2013;39(7):1331–40. 10.1016/j.burns.2013.04.026 23768707

[3] Pereira C, Murphy K, Herndon D. Outcome measures in burn care. Is mortality dead? Burns. 2004;30(8):761–71. 10.1016/j.burns.2004.05.012 15555787

[4] Elmasry M, Steinvall I, Thorfinn J, Abbas AH, Abdelrahman I, Adly OA, et al Treatment of Children With Scalds by Xenografts: Report From a Swedish Burn Centre. J Burn Care Res. 2016. 10.1097/BCR.0000000000000379 27380124

[5] Engrav LH, Heimbach DM, Rivara FP, Kerr KF, Osler T, Pham TN, et al Harborview burns—1974 to 2009. PLoS One. 2012;7(7):e40086 PubMed Central PMCID: PMC3390332. 10.1371/journal.pone.0040086 22792216 PMC3390332

[6] Johnson LS, Shupp JW, Pavlovich AR, Pezzullo JC, Jeng JC, Jordan MH. Hospital length of stay—does 1% TBSA really equal 1 day? J Burn Care Res. 2011;32(1):13–9. 10.1097/BCR.0b013e318204b3ab 21131842

[7] Pavlovich AR, Shupp JW, Jeng JC. Is length of stay linearly related to burn size? A glimmer from the national burn repository. J Burn Care Res. 2009;30(2):229–30. 10.1097/BCR.0b013e318198e77a 19165092

[8] National Burn Repository 2015. Annual Report. American Burn Association. [10 February 2016]. Available from: http://www.ameriburn.org/2015NBRAnnualReport.pdf.

[9] Unal S, Ersoz G, Demirkan F, Arslan E, Tutuncu N, Sari A. Analysis of skin-graft loss due to infection: infection-related graft loss. Annals of plastic surgery. 2005;55(1):102–6. 15985801 10.1097/01.sap.0000164531.23770.60

[10] Gravante G, Delogu D, Esposito G, Montone A. Analysis of prognostic indexes and other parameters to predict the length of hospitalization in thermally burned patients. Burns. 2007;33(3):312–5. 10.1016/j.burns.2006.07.003 17210227

[11] Peck MD, Mantelle L, Ward CG. Comparison of length of hospital stay to mortality rate in a regional burn center. J Burn Care Rehabil. 1996;17(1):39–44. 8808358 10.1097/00004630-199601000-00010

[12] Still J, Donker K, Law E, Thiruvaiyaru D. A program to decrease hospital stay in acute burn patients. Burns. 1997;23(6):498–500. 9429030 10.1016/s0305-4179(97)00044-2

[13] Sjoberg F, Danielsson P, Andersson L, Steinwall I, Zdolsek J, Ostrup L, et al Utility of an intervention scoring system in documenting effects of changes in burn treatment. Burns. 2000;26(6):553–9. 10869827 10.1016/s0305-4179(00)00004-8

[14] Steinvall I, Bak Z, Sjoberg F. Acute respiratory distress syndrome is as important as inhalation injury for the development of respiratory dysfunction in major burns. Burns. 2008;34(4):441–51. 10.1016/j.burns.2007.10.007 18243566

[15] Liffner G, Bak Z, Reske A, Sjoberg F. Inhalation injury assessed by score does not contribute to the development of acute respiratory distress syndrome in burn victims. Burns. 2005;31(3):263–8. 10.1016/j.burns.2004.11.003 15774279

[16] Bak Z, Sjoberg F, Eriksson O, Steinvall I, Janerot-Sjoberg B. Hemodynamic changes during resuscitation after burns using the Parkland formula. J Trauma. 2009;66(2):329–36. 10.1097/TA.0b013e318165c822 19204504

[17] Janzekovic Z. A new concept in the early excision and immediate grafting of burns. J Trauma. 1970;10(12):1103–8. 4921723

[18] Herndon DN, Barrow RE, Rutan RL, Rutan TC, Desai MH, Abston S. A comparison of conservative versus early excision. Therapies in severely burned patients. Ann Surg. 1989;209(5):547–52; discussion 52–3. PubMed Central PMCID: PMC1494069. 2650643 10.1097/00000658-198905000-00006 PMC1494069

[19] Polit DF, Hungler BP. Nursing research Principles and methods. 4th ed. Philadelphia, Pennsylvania: J.B. Lippincott Company; 1991.

[20] Hansbrough W, Dore C, Hansbrough JF. Management of skin-grafted burn wounds with Xeroform and layers of dry coarse-mesh gauze dressing results in excellent graft take and minimal nursing time. J Burn Care Rehabil. 1995;16(5):531–4. 8537426 10.1097/00004630-199509000-00012

[21] Andel D, Kamolz LP, Niedermayr M, Hoerauf K, Schramm W, Andel H. Which of the abbreviated burn severity index variables are having impact on the hospital length of stay? J Burn Care Res. 2007;28(1):163–6. 10.1097/BCR.0B013E31802C9E8F 17211220

[22] Deitch EA, Wheelahan TM, Rose MP, Clothier J, Cotter J. Hypertrophic burn scars: analysis of variables. J Trauma. 1983;23(10):895–8. 6632013

[23] Wong MK, Ngim RC. Burns mortality and hospitalization time—a prospective statistical study of 352 patients in an Asian National Burn Centre. Burns. 1995;21(1):39–46. 7718118 10.1016/0305-4179(95)90780-4

[24] Meshulam-Derazon S, Nachumovsky S, Ad-El D, Sulkes J, Hauben DJ. Prediction of morbidity and mortality on admission to a burn unit. Plast Reconstr Surg. 2006;118(1):116–20. Epub 2006/07/04. 10.1097/01.prs.0000221111.89812.ad 16816682

[25] Saffle JR, Davis B, Williams P. Recent outcomes in the treatment of burn injury in the United States: a report from the American Burn Association Patient Registry. J Burn Care Rehabil. 1995;16(3 Pt 1):219–32; discussion 88–9. 7673300 10.1097/00004630-199505000-00002

[26] Bowser BH, Caldwell FT, Baker JA, Walls RC. Statistical methods to predict morbidity and mortality: self assessment techniques for burn units. Burns Incl Therm Inj. 1983;9(5):318–26. 6871751 10.1016/0305-4179(83)90077-3

[27] Attia AF, Reda AA, Mandil AM, Arafa MA, Massoud N. Predictive models for mortality and length of hospital stay in an Egyptian burns centre. Eastern Mediterranean health journal = La revue de sante de la Mediterranee orientale = al-Majallah al-sihhiyah li-sharq al-mutawassit. 2000;6(5–6):1055–61. 12197328

[28] Ho WS, Ying SY, Burd A. Outcome analysis of 286 severely burned patients: retrospective study. Hong Kong Med J. 2002;8(4):235–9. 12167725

[29] Wearn C, Hardwicke J, Kitsios A, Siddons V, Nightingale P, Moiemen N. Outcomes of burns in the elderly: Revised estimates from the Birmingham Burn Centre. Burns. 2015;41(6):1161–8. Epub 2015/05/20. 10.1016/j.burns.2015.04.008 25983286

