# Supplementary material for: Division of overall duration of stay into operative stay and postoperative stay improves the overall estimate as a measure of quality of outcome in burn care
Source: PLoS One. 2017 Mar 31;12(3):e0174579. doi: 10.1371/journal.pone.0174579 (PMC5376076; doi:10.1371/journal.pone.0174579)
Supplement: S1 Table — A multivariable regression analysis showed that duration of operative stay can be predicted by the extent and depth of the burn injury. The three variables: superficial second degree %, deep second degree %, and full thickness burn %, gave a model adjusted R2 of 0.56. (PDF) [file pone.0174579.s001.pdf]

S1 Table. The predictive model for operative stay

	Coefficient	p value	95% CI
Superficial second degree %	0.4224	0.03	0.04 to 0.80
Deep second degree %	0.5109	<0.001	0.24 to 0.78
Full thickness burn %	2.0430	<0.001	1.78 to 2.31
Constant	2.2731	0.22	-1.34 to 5.89

A multivariable regression analysis showed that duration of operative stay can be predicted by the extent and depth of the burn injury. The three variables: superficial second degree %, deep second degree %, and full thickness burn %, gave a model adjusted R^2 of 0.56. ($p<0.001$, n=217)
